# Hyperthyroidism or hypothyroidism and gastrointestinal cancer risk: a Danish nationwide cohort study

**DOI:** 10.1530/EC-18-0258

**Published:** 2018-08-31

**Authors:** Jakob Kirkegård, Dora Körmendiné Farkas, Jens Otto Lunde Jørgensen, Deirdre P Cronin-Fenton

**Affiliations:** 1 Department of Clinical EpidemiologyAarhus University Hospital, Aarhus N, Denmark; 2 Department of Surgery (Section for Upper Gastrointestinal and Hepatico-Pancreatico-Biliary Surgery)Aarhus University Hospital, Aarhus N, Denmark; 3 Department of EndocrinologyAarhus University Hospital, Aarhus N, Denmark

**Keywords:** thyroid disease, hyperthyroidism, hypothyroidism, gastrointestinal cancer, risk factor, epidemiology

## Abstract

**Objective:**

The association between thyroid dysfunction and gastrointestinal cancer is unclear.

**Design:**

We conducted a nationwide population-based cohort study to examine this potential association.

**Methods:**

We used Danish medical registries to assemble a nationwide population-based cohort of patients diagnosed with hyperthyroid or hypothyroid disease from 1978 to 2013. We computed standardized incidence ratios (SIRs) with corresponding 95% CIs as measures of the relative risk of each cancer, comparing patients with thyroid dysfunction with that expected in the general population.

**Results:**

We included 163,972 patients, of which 92,783 had hyperthyroidism and 71,189 had hypothyroidism. In general, we found an increased risk of all gastrointestinal cancers within the first year after thyroid disease diagnosis. After more than 5 years of follow-up, patients with hyperthyroidism had a slightly increased risk of pancreatic and gallbladder and biliary tract cancer. Patients with hypothyroidism had a slightly increased risk of stomach, anal, liver, gallbladder and biliary tract, and pancreatic cancer after more than 5 years of follow-up, but the observed numbers of cancers were in general similar to the expected.

**Conclusions:**

The increased risks of all gastrointestinal cancers in the first year following hyper- or hypothyroidism diagnosis are likely due to detection bias. After more than 5 years of follow-up, there does not seem to be a consistent causal association between thyroid disease and gastrointestinal cancer.

## Introduction

Iodothyronines secreted from the thyroid gland – in particular triiodothyronine (T3) and thyroxine (T4) – are vital to the regulation of genes associated with cell metabolism and cell growth (1). Hyperthyroidism and hypothyroidism are defined by an excess or deficiency of T3 and T4, respectively and can affect organ function and increase mortality (2).

Thyroid hormone status affects the growth and homeostasis of gastrointestinal organs through binding to thyroid hormone receptors in the gastrointestinal epithelium (3). Despite this, information on the association of hyperthyroid or hypothyroid disease with gastrointestinal cancer incidence is sparse. A cohort study of more than 7000 women revealed a higher incidence of pancreatic cancer in hyperthyroid patients compared with their euthyroid counterparts (4). A case–control study of ~1500 patients suggested an elevated risk of liver cancer associated with hypothyroid disease (5). Furthermore, in a case–control study of 262 individuals, more cases of hyperthyroidism were observed among esophageal cancer cases compared with their controls (6). In addition, T4 substitution therapy has been correlated with a decreased risk of colorectal cancer (7).

Thus, the association of hyperthyroidism and hypothyroidism diagnoses with the risk of gastrointestinal cancer has been evaluated for some, but not all, gastrointestinal cancers. We therefore conducted a population-based cohort study to examine the association between a diagnosis of hyperthyroid or hypothyroid disease and the incidence of gastrointestinal cancer overall and according to cancer site.

## Subjects and methods

We conducted a nationwide population-based Danish cohort study between January 1, 1978 and November 30, 2013. Individual-level data linkage of Danish medical registries was possible using the civil registration number, a unique identification number assigned to all Danish residents at birth or immigration (8).

### Study population

From the Danish National Patient Registry, we identified all patients with a hospital diagnosis of hyperthyroidism or hypothyroidism in Denmark during the study period. The Danish National Patient Registry has registered all inpatient hospital admissions since 1977 and all outpatient clinic and emergency department visits since 1995 (9). In the Danish National Patient Registry, all diagnoses are classified according to the 8th revision of the *International Classification of Diseases* (ICD-8) until 1994 and the 10th revision (ICD-10) thereafter. The ICD codes used in this study are listed in Supplementary Table 1 (see section on supplementary data given at the end of this article). We excluded all patients born outside Denmark (*n* = 12,695) and patients diagnosed with hyperthyroidism and hypothyroidism on the same date (*n* = 537).

### Data on cancers and comorbid conditions

Using the Danish Cancer Registry (DCR) we retrieved data on gastrointestinal cancer diagnoses. The DCR contains data on almost all cancer diagnoses in Denmark since 1943 (10). We considered the following cancers: esophageal, stomach, small intestinal, colon, rectal, anal, liver, gallbladder and biliary tract and pancreatic cancer. We excluded all patients diagnosed with these cancers before the date of diagnosis of hyperthyroidism or hypothyroidism. We also grouped the cancers into groups based on common etiologies: alcohol-related (esophageal, small intestinal, colon, rectum, liver), smoking-related (esophageal, stomach, colon, rectum and pancreas), immune-related (stomach, small intestinal, anal, liver and gallbladder and biliary tract) and obesity-related (esophageal, stomach, colon, rectum, pancreas and gallbladder and biliary tract) cancers (11, 12, 13, 14, 15, 16, 17, 18). When assessing the association of thyroid disease with rectal cancer risk, we ascertained information on the receipt of a colonoscopy with or without polyp removal after diagnosis of thyroid disease disregarding colonoscopies performed within 1 month before rectal cancer diagnosis. Information on colonoscopies was retrieved from the Danish National Patient Registry using the Nordic Medico-Statistical Committee Classification of Surgical Procedures (See Supplementary Table 1 for procedure codes) (19).

To assess comorbid conditions recorded in the Danish National Patient Registry, we used the Charlson Comorbidity Index (CCI) score (20), which is based on disease categories, each weighted according to its impact on 1-year mortality. The CCI can be used to assess the overall burden of comorbidity (21). We defined three levels of comorbidity: no (score 0), moderate (score 1–2) and severe comorbidity (score ≥ 3). We excluded gastrointestinal cancers from the CCI score. We also calculated the prevalence of some selected comorbid conditions likely to confound our findings (alcoholism, inflammatory bowel disease, other autoimmune disease, pancreatitis, chronic obstructive pulmonary disease (COPD), diabetes, obesity, human immunodeficiency virus (HIV) and gastrointestinal bleeding).

### Statistical analyses

We followed all patients from the date of their thyroid disease diagnosis until the date of the first gastrointestinal cancer diagnosis, emigration, death or November 30, 2013, whichever occurred first. Patients with hyperthyroidism were censored if they had a subsequent diagnosis of hypothyroidism and vice versa. We calculated standardized incidence ratios (SIRs) as a measure of the relative risk, comparing the observed number of cancers among patients with hyperthyroidism or hypothyroidism with the expected number. The expected number of cancers was estimated based on national cancer incidence rates by age (1-year age groups), sex and calendar year (1-year intervals) multiplied by the time of follow-up observed in our cohort. We computed corresponding 95% CIs for the SIRs assuming that the observed number of cases in a specific category followed a Poisson distribution. Exact 95% CIs were used when the observed number was less than ten; otherwise Byar’s approximation was used (22). We performed analyses for each type of cancer stratified by duration of follow-up (<1, 1–5, >5 years and overall) and type of thyroid disease. We computed 10-year absolute risks of the gastrointestinal cancers of interest, treating death as a competing risk.

### Ethical considerations

This study was approved by the Danish Data Protection Agency (record number 1-16-02-1-08). No ethical approval or patient consent is needed for registry-based studies conducted in Denmark.

## Results

### Overall patient characteristics

We included 163,972 patients; 92,783 patients had hyperthyroidism and 71,189 had hypothyroidism. In both groups, the vast majority of patients were women (82.5 and 83.9% respectively). In general, patients with hypothyroidism had a higher level of comorbidity compared with hyperthyroid patients. Hypothyroid patients were more likely to have a history of obesity, COPD, autoimmune diseases and diabetes. There was an equal distribution of in- and outpatient diagnoses among both hyperthyroidism and hypothyroidism patients (Table 1). Descriptive characteristics stratified by type of hyperthyroidism are outlined in Table 2.

**Table 1:** Descriptive characteristics of 163,972 patients with hyper- or hypothyroidism diagnosed in Denmark in the period 1978–2013.

	Hyperthyroidism *N* = 92,783		Hypothyroidism *N* = 71,189	
Age (years)				
0–29	6552	7.1%	6147	8.6%
30–49	21,507	23.2%	15,291	21.5%
50–69	34,490	37.2%	23,360	32.8%
70+	30,234	32.6%	26,391	37.1%
Sex				
Women	76,564	82.5%	59,721	83.9%
Men	16,219	17.5%	11,468	16.1%
Year of diagnosis				
1978–1982	8015	8.6%	3651	5.1%
1983–1987	6828	7.4%	3815	5.4%
1988–1992	7728	8.3%	5024	7.1%
1993–1997	14,184	15.3%	7612	10.7%
1998–2002	18,501	20.0%	10,766	15.1%
2003–2007	18,458	19.9%	15,413	21.7%
2008–2013	19,069	20.6%	24,908	35.0%
Patient type				
Inpatient	43,482	47.3%	35,762	50.2%
Half-day patient	3855	4.2%	1979	2.8%
Outpatient	45,086	48.6%	33,448	47.0%
Comorbidity				
Alcoholism	1338	1.4%	2048	2.9%
Autoimmune disease	6970	7.5%	8865	12.5%
Pancreatitis	593	0.6%	728	1.0%
COPD	7765	8.4%	7269	10.2%
IBD	935	1.0%	847	1.2%
Colitis ulcerosa	619	0.7%	553	0.8%
Crohn’s disease	338	0.4%	273	0.4%
Diabetes	6102	6.6%	7671	10.8%
Obesity	3069	3.3%	5341	7.5%
HIV	22	<0.1%	20	<0.1%
GI bleeding	764	0.8%	1023	1.4%
Charlson Comorbidity Index score				
Low (0)	69,852	75.3%	46,643	68.3%
Medium (1–2)	19,663	21.2%	18,676	26.2%
High (≥3)	3268	3.5%	3870	5.4%

COPD, chronic obstructive pulmonary disease; GI, gastrointestinal; HIV, human immunodeficiency virus; IBD, inflammatory bowel disease.

**Table 2:** Descriptive characteristics of 92,783 patients with hyperthyroidism diagnosed in Denmark in the period 1978–2013, stratified by type of hyperthyroidism.

	Hyperthyroidism *N* = 92,783		
	**Graves’ disease** *N* (%)	**Struma nodosa toxica** *N* (%)	**Other hyperthyroidism** *N* (%)
Total	38,508 (100.0)	34,703 (100.0)	19,572 (100.0)
Age (years)			
0–29	4149 (10.8)	922 (2.7)	1481 (7.6)
30–49	11,649 (30.3)	5844 (16.8)	4014 (20.5)
50–69	13,120 (34.1)	14,897 (42.9)	6473 (33.1)
70+	9590 (24.9)	13,040 (37.6)	7604 (38.9)
Sex			
Women	31,897 (82.8)	29,166 (84.0)	15,501 (79.2)
Men	6611 (17.2)	5537 (16.0)	4071 (20.8)
Year of diagnosis			
1978–1982	3082 (8.0)	4932 (14.2)	1 (<0.1)
1983–1987	2353 (6.1)	4475 (12.9)	0 (0.0)
1988–1992	2601 (6.8)	5022 (14.4)	105 (0.5)
1993–1997	6385 (16.6)	4993 (14.4)	2806 (14.3)
1998–2002	8649 (22.5)	5395 (15.6)	4457 (22.8)
2003–2007	8975 (23.3)	4765 (13.7)	4718 (24.1)
2008–2013	6463 (16.8)	5121 (14.8)	7485 (38.2)
Patient type			
Inpatient	17,730 (46.0)	18,240 (52.6)	7872 (40.2)
Half-day patient	1686 (4.4)	1680 (4.8)	489 (2.5)
Outpatient	19,092 (49.6)	14,783 (42.6)	11,211 (57.3)
Comorbidity			
Alcoholism	583 (1.5)	318 (0.9)	437 (2.2)
Autoimmune disease	2771 (7.2)	2270 (6.5)	1929 (9.9)
Pancreatitis	214 (0.6)	186 (0.5)	193 (1.0)
COPD	2830 (7.4)	2653 (7.7)	2281 (11.7)
IBD	381 (1.0)	292 (0.8)	262 (1.3)
Colitis ulcerosa	259 (0.7)	183 (0.5)	177 (0.9)
Crohn’s disease	138 (0.4)	102 (0.3)	98 (0.5)
Diabetes	2226 (5.8)	2321 (6.7)	1555 (8.0)
Obesity	1194 (3.1)	965 (2.8)	910 (4.7)
HIV	7 (<0.1)	5 (<0.1)	10 (0.1)
GI bleeding	273 (0.7)	223 (0.6)	268 (1.4)
Charlson Comorbidity Index score			
Low (0)	30,187 (78.4)	26,670 (76.9)	12,995 (66.4)
Medium (1–2)	7210 (18.7)	7061 (20.3)	5392 (27.6)
High (≥3)	1111 (2.9)	972 (2.8)	1185 (6.1)

COPD, chronic obstructive pulmonary disease; GI, gastrointestinal; HIV, human immunodeficiency virus; IBD, inflammatory bowel disease.

### Hyperthyroid patients (*n* = 92,783)

Patients were followed for a median time of 7.5 years (inter-quartile range (IQR): 3.0–13.4 years). Median age at hyperthyroid diagnosis was 61.0 years (IQR: 46.5–73.7 years). In patients with hyperthyroid disease, we observed an increased risk of both overall gastrointestinal cancer (SIR: 1.17; 95% CI: 1.13–1.22) and site-specific gastrointestinal cancers except cancer of the small intestines (Table 3). Within the first year following a diagnosis of hyperthyroid disease, risk of all gastrointestinal cancers was elevated. After more than 5 years of follow-up, only pancreatic and gallbladder and biliary tract cancer risk was slightly elevated (Table 3). Results stratified by type of hyperthyroid disorder are presented in Supplementary Tables 2, 3 and 4. Overall, the absolute risk of gastrointestinal cancer was 2.4% (95% CI: 2.3–2.5%) after 10 years of follow-up.

**Table 3:** SIRs and associated 95% CIs for gastrointestinal cancers in 92,783 patients with hyperthyroidism diagnosed in Denmark in the period 1978–2013, stratified by time of follow-up.

Cancer site	Overall			<1 year			1–5 years			>5 years		
	**O**	**E**	**SIR**	**O**	**E**	**SIR**	**O**	**E**	**SIR**	**O**	**E**	**SIR**
Overall	2628	2246	1.17 (1.13–1.22)	480	208	2.31 (2.10–2.52)	726	687	1.06 (0.98–1.14)	1422	1351	1.05 (1.00–1.11)
Esophagus	117	103	1.14 (0.94–1.37)	25	9	2.67 (1.73–3.94)	24	31	0.77 (0.49–1.16)	68	62	1.09 (0.85–1.39)
Stomach	215	174	1.24 (1.08–1.42)	45	18	2.50 (1.83–3.35)	62	57	1.08 (0.83–1.39)	108	98	1.10 (0.90–1.33)
Small intestines	29	28	1.04 (0.70–1.50)	5	2	2.04 (0.66–4.75)	9	8	1.09 (0.50–2.08)	15	17	0.87 (0.49–1.44)
Colon	1122	1005	1.12 (1.05–1.18)	203	91	2.22 (1.93–2.55)	303	304	1.00 (0.89–1.12)	616	609	1.01 (0.93–1.09)
Rectum	484	427	1.13 (1.04–1.24)	73	40	1.83 (1.43–2.30)	147	131	1.12 (0.95–1.32)	264	255	1.03 (0.91–1.17)
Anal canal	44	37	1.18 (0.86–1.58)	11	3	3.50 (1.74–6.26)	9	11	0.84 (0.38–1.59)	24	23	1.03 (0.66–1.53)
Liver	90	83	1.08 (0.87–1.33)	18	8	2.27 (1.34–3.59)	18	26	0.70 (0.41–1.10)	54	50	1.09 (0.82–1.42)
Gallbladder and biliary tract	102	82	1.25 (1.02–1.51)	16	8	2.00 (1.14–3.25)	30	26	1.16 (0.78–1.66)	56	48	1.17 (0.88–1.51)
Pancreas	425	308	1.38 (1.25–1.52)	84	28	3.00 (2.39–3.71)	124	93	1.33 (1.11–1.59)	217	187	1.16 (1.01–1.32)
Smoking-related cancers	2363	2016	1.17 (1.13–1.22)	430	187	2.30 (2.09–2.53)	660	617	1.07 (0.99–1.16)	1273	1213	1.05 (0.99–1.11)
Immune-related cancers	480	404	1.19 (1.08–1.30)	95	40	2.40 (1.95–2.94)	128	128	1.00 (0.84–1.19)	257	237	1.09 (0.96–1.23)
Alcohol-related cancers	1842	1645	1.12 (1.07–1.17)	324	151	2.15 (1.92–3.29)	501	500	1.00 (0.92–1.09)	1017	994	1.02 (0.96–1.09)
Obesity-related cancers	2465	2098	1.18 (1.13–1.22)	446	195	2.29 (2.08–2.51)	690	643	1.07 (1.00–1.16)	1329	1261	1.05 (1.00–1.11)

E, expected events; O, observed events; SIR, standardized incidence ratio.

### Hypothyroid patients (*n* = 71,189)

Patients were followed for a median time of 4.9 years (IQR: 1.8–9.9 years). Median age at hypothyroid diagnosis was 62.9 years (IQR: 45.9–76.1 years). Patients with hypothyroidism had an increased risk of gastrointestinal cancer overall (SIR: 1.23; 95% CI: 1.17–1.30) and increased risk of each gastrointestinal cancer except for rectal cancer (Table 4). Within the first year following a diagnosis of hypothyroid disease, risk of all gastrointestinal cancers was elevated. The risk of cancer of the stomach, anal canal, liver, gallbladder and biliary tract and immune related was increased beyond 5 years of follow-up. The risk of rectal cancer was increased within the first year after hypothyroidism diagnosis but was decreased after 5 years (SIR: 0.79; 95% CI: 0.64–0.96). In total, 11,232 patients (15.8%) underwent colonoscopy prior to their diagnosis of hypothyroidism. Patients who received a colonoscopy with polyp removal had lower risk of rectal cancer compared with the background population (SIR: 0.29; 95% CI: 0.01–1.63). In contrast, the risk of rectal cancer among patients undergoing colonoscopy without polyp removal was comparable to that in the general population (SIR: 1.09; 95% CI: 0.68–1.67). The absolute risk of gastrointestinal cancer overall was 2.4% (95% CI: 2.2–2.5) after 10 years of follow-up.

**Table 4:** SIRs and associated 95% CIs for gastrointestinal cancers in 71,189 patients with hypothyroidism diagnosed in Denmark in the period 1978–2013, stratified by time of follow-up.

Cancer site	Overall			<1 year			1–5 years			>5 years		
	**O**	**E**	**SIR**	**O**	**E**	**SIR**	**O**	**E**	**SIR**	**O**	**E**	**SIR**
Overall	1608	1303	1.23 (1.17–1.30)	502	165	3.04 (2.78–3.32)	449	479	0.94 (0.85–1.03)	657	659	1.00 (0.92–1.08)
Esophagus	76	57	1.32 (1.04–1.66)	26	7	3.59 (2.35–5.27)	19	21	0.90 (0.54–1.41)	31	29	1.07 (0.73–1.51)
Stomach	148	99	1.49 (1.26–1.75)	43	14	3.12 (2.26–4.21)	44	39	1.14 (0.83–1.53)	61	47	1.30 (0.99–1.66)
Small intestines	20	16	1.25 (0.77–1.94)	9	2	4.60 (2.11–8.75)	5	6	0.86 (0.28–2.02)	5	8	0.73 (0.27–1.59)
Colon	731	591	1.24 (1.15–1.33)	230	74	3.12 (2.73–3.55)	215	215	1.00 (0.87–1.14)	286	302	0.95 (0.84–1.06)
Rectum	232	243	0.95 (0.83–1.08)	75	31	2.41 (1.90–3.03)	61	90	0.68 (0.52–0.87)	96	122	0.79 (0.64–0.96)
Anal canal	30	22	1.39 (0.94–1.99)	6	3	2.36 (0.86–5.13)	5	8	0.65 (0.21–1.51)	19	11	1.68 (1.01–2.63)
Liver	71	47	1.50 (1.17–1.89)	19	6	3.11 (1.87–4.86)	19	18	1.08 (0.65–1.69)	31	24	1.39 (0.96–1.96)
Gallbladder and biliary tract	72	48	1.49 (1.17–1.88)	19	6	3.01 (1.81–4.70)	20	18	1.11 (0.68–1.71)	33	24	1.38 (0.95–1.94)
Pancreas	228	179	1.27 (1.11–1.45)	75	22	3.36 (2.64–4.21)	61	65	0.93 (0.71–1.20)	92	92	1.00 (0.81–1.23)
Smoking-related cancers	1415	1170	1.21 (1.15–1.27)	449	148	3.03 (2.76–3.33)	400	430	0.93 (0.84–1.03)	566	592	0.96 (0.88–1.04)
Immune-related cancers	341	233	1.47 (1.32–1.63)	96	31	3.13 (2.53–3.82)	93	88	1.06 (0.86–1.30)	152	114	1.33 (1.13–1.56)
Alcohol-related cancers	1130	955	1.18 (1.12–1.25)	359	120	2.99 (2.69–3.32)	319	350	0.91 (0.82–1.02)	452	485	0.93 (0.85–1.02)
Obesity-related cancers	1487	1218	1.22 (1.16–1.28)	468	154	3.03 (2.76–3.32)	420	448	0.94 (0.85–1.03)	599	616	0.97 (0.90–1.05)

E, expected events; O, observed events; SIR, standardized incidence ratio.

## Discussion

In this cohort study of 163,972 Danish patients with a hospital-verified diagnosis of either hyperthyroidism or hypothyroidism, we found an elevated risk of most gastrointestinal cancers compared with the general population. The associations were most pronounced in the first year following hyper- or hypothyroidism diagnosis. Although the associations generally attenuated over time, an excess risk of some gastrointestinal cancers may persist more than 5 years after a diagnosis of either hyperthyroidism or hypothyroidism.

For all associations examined in the present study, gastrointestinal cancer risks were highest in the first year following a diagnosis of either hyperthyroidism or hypothyroidism. This is likely to be attributable to increased medical attention (i.e. detection bias) due to frequent contact to the healthcare system in the first period following a diagnosis of a thyroid disease. Some gastrointestinal cancers may also present with symptoms mimicking thyroid dysfunction such as fatigue, fever, gastrointestinal symptoms and weight loss. This could lead to an early cancer diagnosis, increasing the SIRs in the first year of follow-up (i.e. reverse causation).

Few studies have investigated the long-term risk of gastrointestinal cancer in patients with thyroid disease (4, 5, 6, 7). Our estimates of the association between hyperthyroidism and gastrointestinal cancer risk after more than 5 years of follow-up were generally distributed around the null, indicating no excess or decreased cancer risk. However, we observed slight increases in pancreatic, and gallbladder and biliary tract cancer among patients with hyperthyroidism. Similarly, Goldman *et al.* (4) demonstrated an increased risk of pancreatic cancer in patients with hyperthyroidism, although their estimates were imprecise due to low numbers. Confounding by tobacco smoking may explain the observed increase in pancreatic cancer risk among patients with hyperthyroidism, as tobacco smoking is associated with both hyperthyroidism (23) and pancreatic cancer (15). Unfortunately, tobacco smoking is not recorded in the Danish National Patient Registry. Accordingly, we were unable to control for this factor. However, we saw no increase in the risk of smoking-related cancers as a group among patients with hyperthyroidism, reducing the likelihood that tobacco smoking is the sole explanation for this observed association.

Among patients with hypothyroidism, the numbers of observed and expected cancers were comparable, with the exception of cancers of the stomach, rectum and anus. Our finding of an increased risk of stomach cancer agrees with results from Goldman *et al.* (4) who observed a higher incidence of stomach cancer in patients with Hashimoto’s thyroiditis – a subtype of hypothyroidism. As tobacco smoking is a risk factor for both stomach cancer (18) and Hashimoto’s thyroiditis, confounding by tobacco smoking could be an explanation for our finding, although we did not observe an increased risk of other smoking-related cancers among patients with hypothyroid disease. The observed increased risk of anal cancer in patients with hypothyroidism is based on low numbers of cancers and is therefore most likely attributable to chance.

In the present study, we found a slight decrease in long-term rectal cancer risk among patients with hypothyroidism. Hypothyroidism may cause gastrointestinal bleeding (24), leading to colonoscopies, thereby decreasing rectal cancer risk via removal of rectal polyps. Accordingly, we observed a decreased risk of rectal cancer in patients undergoing colonoscopy with polyp removal but not among those without polyp removal, compared with the background population. However, our estimates of this association are imprecise.

Several issues should be considered when interpreting our findings. The population-based design of our study in a tax-financed uniform health care system ensured equal medical access for all participants and long-term, virtually complete follow-up. The prospective data collection from valid electronic registries minimizes the possibilities of underreporting of cancer diagnoses or misclassification of exposure. Nonetheless, we had no access to primary care data, and therefore, no information on subclinical thyroid disease, which is more common than clinical thyroid disease (25). Accordingly, our observed associations may be overestimated, as any potential association between thyroid disease and gastrointestinal cancer may depend on the severity of the thyroid disease. We had no data on potential confounders such as environmental or lifestyle exposures including, among others, tobacco smoking and alcohol consumption. We also lacked information on blood levels of thyroid-stimulating hormone and thyroid hormones, as well as data on prescription drugs indicated for thyroid disease. Prompt medical treatment after the diagnosis of a thyroid disorder is likely to reverse the effect of thyroid disease, which may neutralize an association between thyroid disease and gastrointestinal cancer risk. Furthermore, in patients with anal and small intestinal cancer, the observed number of cancers was very small, leading to imprecise estimates.

In conclusion, our observed increased risk of all gastrointestinal cancers within the first year after thyroid disease diagnosis is likely attributable to reverse causation or detection bias. There does not seem to be a causal association between thyroid disease and long-term risk of gastrointestinal cancer.

## Supplementary Material

Supporting Table 1

Supporting Table 2

Supporting Table 3

Supporting Table 4

## Declaration of interest

Jens Otto Lunde Jørgensen is an editor of the journal. The other authors have no conflicts of interest.

## Funding

This work was supported by the Program for Clinical Research Infrastructure, established by the Lundbeck and the Novo Nordisk Foundations; by the Danish Cancer Society (R73-14284-13-S17); by the Aarhus University Research Foundation and by the Danish Medical Research Council (DFF-4183-00359). The funders had no role in conducting this study.

## Author contribution statement

J K contributed to data interpretation, paper drafting and critical revisions. J O L J helped in data interpretation and critical revisions. D K F contributed to study design, data analysis and interpretation and critical revisions. D P C-F helped in study design, data interpretation, paper drafting and critical revisions. All authors approved the manuscript and agreed to be accountable for all aspects of the work.
