# Supplementary material for: Hyperthyroidism or hypothyroidism and gastrointestinal cancer risk: a Danish nationwide cohort study
Source: Endocr Connect. 2018 Aug 31;7(11):1129–35. doi: 10.1530/EC-18-0258 (PMC6215792; doi:10.1530/EC-18-0258)
Supplement: Supporting Table 1 [file ec-7-1129-t001.pdf]

ELECTRONIC SUPPLEMENTARY MATERIAL

Manuscript title: Hyper- and Hypothyroidism and Gastrointestinal Cancer Risk: A Danish nationwide cohort study

Journal: European Journal of Endocrinology

Authors: Jakob Kirkegård[1,2]**,** Dora Körmendiné Farkas[1], Jens Otto Lunde Jørgensen[3], Deirdre P. Cronin-Fenton[1]

[1] Department of Clinical Epidemiology, Aarhus University Hospital, Olof Palmes Allé 43-45, 8200 Aarhus N, Denmark

[2] Department of Surgery (section for upper gastrointestinal and hepatico-pancreatico-biliary surgery), Aarhus University Hospital, Nørrebrogade 44, 8000 Aarhus C, Denmark

[3] Department of Endocrinology, Nørrebrogade 44, 8000 Aarhus C, Aarhus University Hospital, Denmark

Corresponding author address:

Jakob Kirkegård, Department of Surgery, Aarhus University Hospital, Palle Juul-Jensens Boulevard 99, DK-8200 Aarhus N, Denmark

Phone: +45 87 46 00 00 / E-mail: jakob.kirkegaard@auh.rm.dk

Supplementary Table 1. International Classification of Diseases (ICD)-8 and -10 codes.

**Hyperthyroid disease**	
Graves' disease	ICD-8: 242.0; ICD-10: DE05.0
Struma nodosa toxica	ICD-8: 242.1, 242.2, 242.3; ICD-10: DE05.1, DE05.2
Other hyperthyroidism	ICD-8: 242.4-242.9; ICD-10: DE05.3-DE05.9
**Hypothyroid disease**	
Hashimoto's thyroiditis	ICD-8: 245.2; ICD-10: DE06.3
Other hypothyroidism	ICD-8: 244; ICD-10: DE03.2-DE03.9; DE89.0
**Cancer diagnoses**	
Esophagus	ICD-8: 150; ICD-10: C15
Stomach	ICD-8: 151; ICD-10: C16
Small intestine	ICD-8: 152; ICD-10: C17
Colon	ICD-8: 153, 154.0; ICD-10: C18, C19
Rectum	ICD-8: 154.1; ICD-10: C20
Anal canal	ICD-8: 154.2 ICD-10: C21
Liver	ICD-8: 155; ICD-10: C22
Gallbladder and biliary tract	ICD-8: 156; ICD-10: C23, C24
Pancreas	ICD-8: 157; ICD-10: C25
**Charlson Comorbidity Index**	
Score 1	
Myocardial infarction	ICD-8: 410; ICD-10: I21, I22, I23
Congestive heart failure	ICD-8: 427.09, 427.10, 427.11, 427.19, 428.99, 782.49; ICD-10: I50, I11.0, I13.0, I13.2
Peripheral vascular disease	ICD-8: 440, 441, 442, 443, 444, 445; ICD-10: I70, I71, I72,

	I73, I74, I77
Cerebrovascular disease	ICD-8: 430-438; ICD-10: I60-I69, G45, G46
Dementia	ICD-8: 290.09-290.19, 293.09; ICD-10: F00-F03, F05.1, G30
Chronic pulmonary disease	ICD-8: 490-493, 515-518, ICD-10: J40-J47, J60-J67, J68.4, J70.1, J70.3, J84.1, J92.0, J96.1, J98.2, J98.3
Connective tissue disease	ICD-8: 712, 716, 734, 446, 135.99; ICD-10: M05, M06, M08, M09, M30, M31, M32, M33, M34, M35, M36, D86
Ulcer disease	ICD-8: 530.91, 530.98, 531-534; ICD-10: K22.1, K25-K28
Mild liver disease	ICD-8: 571, 573.01, 573.04; ICD-10: B18, K70.0-K70.3, K70.9, K71, K73, K74, K76.0
Diabetes types 1 and 2	ICD-8: 249.00, 249.06, 249.07, 249.09, 250.00, 250.06, 250.07, 250.09; ICD-10: E10.0, E10.1, E10.9, E11.0, E11.1, E11.9
Score 2	
Hemiplegia	ICD-8: 344; ICD-10: G81, G82
Moderate to severe renal disease	ICD-8: 403, 404, 580-583, 584, 590.09, 593.19, 753.10-753.19, 792; ICD-10: 12, I13, N00-N05, N07, N11, N14, N17-N19, Q61
Diabetes with end-organ damage	ICD-8: 249.01-249.05, 249.08, 250.01-250.05, 250.08; ICD-10: E10.2-E10.8, E11.2-E11.8
Any tumor	ICD-8: 140-194, 173.09-173.49, 173.60-173.61, 173.69-99; ICD-10: C00-C75
Leukemia	ICD-8: 204-207; ICD-10: C91-C95
Lymphoma	ICD-8: 200-203, 275.59; ICD-10: C81-C85, C88, C90, C96
Score 3	
Moderate to severe liver disease	ICD-8: 070.00, 070.02, 070.04, 070.06, 070.08, 573.00, 456.00-456.09; ICD-10: B15.0, B16.0, B16.2, B19.0,

	K70.4, K72, K76.6, I85
Score 6	
Metastatic solid tumor	ICD-8: 195-198, 199; ICD-10: C76-C80
AIDS	ICD-8: 079.83; ICD-10: B21-B24
**Comorbid conditions**	
Alcoholism	ICD-8: 291, 303; ICD10: F10
Inflammatory bowel disease	ICD-8: 563; ICD-10: K50, K51, M07.4, M07.5
Mb. Chron	ICD-8: 563.00-563.99, ICD-10: K50, M074
Colitis ulcerosa	ICD-8: 563.19; ICD-10: K51, M07.5
Diabetes	ICD-8: 249, 250; ICD-10: E10-E14
HIV	ICD-8: 079.83; ICD-10: B20-B24, F024
Obesity	ICD-8: 277.99; ICD-10: E65-E66
Gastrointestinal bleeding	ICD-8: 569.15; ICD-10: K62.5
Pancreatitis	ICD-8: 577.0, 577.1; ICD-10: K85, K86.0, K86.1
Other autoimmune conditions	See below
Chronic obstructive pulmonary disease	ICD-8: 490-493, 515-518; ICD-10: J40-J47, J60-J67, J68.4, J70.1, J70.3, J84.1, J92.0, J96.1, J98.2, J98.3
**Procedure codes**	
Colonoscopy	91070, 91071, 91075, 91080, 91081, 91085, 91090, 91091, 91095, 93200, 93210
	KUJF3, KUJF4, KUJG
**Other autoimmune conditions**	
Autoimmune hemolytic anemia	ICD-8: 283.90, 283.91; ICD-10: D59.0, D59.1
Idiopatic trombocytopenic	ICD-8: 287.10; ICD-10: D69.3

purpura	
Grave’s disease	ICD-8: 242.00, 242.01, 242.08, 242.09; ICD-10: E05.0
Autoimmune thyroiditis	ICD-8: 244.01, 245.03; ICD-10: E06.3
Addison´s disease	ICD-8: 255.10, 255.11; ICD-10: E27.1A, E27.2A
Diabetes I	ICD-8: 249; ICD-10: E10
Multiple sclerosis	ICD-8: 340; ICD-10: G35
Myasthenia gravis	ICD-8: 733.09; ICD-10: G70.0
Pernicious anemia	ICD-8: 281.00, 281.01, 281.08, 281.09; ICD-10: D51.0
Coeliac disease	ICD-8: 269.00; ICD-10: K90.0
Crohn´s disease	ICD-8: 563.01, 563.02, 563.09; ICD-10: K50, M07.4
Ulcerative colitis	ICD-8: 563.19; ICD-10: K51, M07.5
Primary biliary cirrhosis	ICD-8: 571.90; ICD-10: K74.3
Pemphigus / pemphigoid	ICD-8: 694.00-694.03, 694.05; ICD-10: L10.0, L10.2, L10.4, L12.0
Dermatitis herpetiformis	ICD-8: 693.08, 693.09; ICD-10: L13.0
Psoriasis	ICD-8: 696.09, 696.10, 696.19; ICD-10: L40, M07.0-M07.3
Vitiligo	ICD-8: 709.01; ICD-10: L80
Scleroderma	ICD-8: 734.0; ICD-10: M34
Juvenile rheumatoid arthritis	ICD-8: 734.0; ICD-10: M05, M06, M08, G73.7D
Rheumatoid arthritis	ICD-8: 712.19, 712.29, 712.39, 712.59; ICD-10: I32.8A, I39.8E, I41.8A, I52.8A
Ankylosing spondylitis	ICD-8: 712.49; ICD-10: M45
Polymyositis / dermatomyositis	ICD-8: 716.09, 716.19; ICD-10: M33
Systemic lupus erythematosus	ICD-8: 734.19; ICD-10: M32, G73.7C, I39.8C, N08.5A,

	N16.4B
Sjögren´s syndrome	ICD-8: 734.90; ICD-10: M35, G73.7A, N16.4A
Sarcoidosis	ICD-8: 135.99; ICD-10: D86, G53.2, H22.1A, I41.8B, K77.8B, M63.3
Polyarteritis nodosa	ICD-8: 446.09; ICD-10: M30.0
Wegener´s granulomatosis	ICD-8: 446.29; ICD-10: M31.3
Temporal arteritis / polymyalgia rheumatica	ICD-8: 446.30, 446.31, 446.39; ICD-10: M31.5, M31.6, M35.3
