# Supplementary material for: Hyperthyroidism or hypothyroidism and gastrointestinal cancer risk: a Danish nationwide cohort study
Source: Endocr Connect. 2018 Aug 31;7(11):1129–35. doi: 10.1530/EC-18-0258 (PMC6215792; doi:10.1530/EC-18-0258)
Supplement: Supporting Table 2 [file ec-7-1129-t002.pdf]

Supplementary Table 2. SIRs for gastrointestinal cancers in 38,508 patients with Graves' disease diagnosed in Denmark in the period 1978-2013, stratified by time of follow-up. Numbers in parentheses indicates 95% CIs.

**Cancer site**	**Overall**			**<1 year**			**1-5 years**			**>5 years**		
	**O**	**E**	**SIR**	**O**	**E**	**SIR**	**O**	**E**	**SIR**	**O**	**E**	**SIR**
Overall	926	837	1.11 (1.04-1.18)	157	70	2.25 (1.91-2.63)	239	242	0.99 (0.86-1.12)	530	525	1.01 (0.93-1.10)
Esophagus	51	39	1.29 (0.96-1.70)	8	3	2.48 (1.07-4.88)	7	11	0.62 (0.25-1.27)	36	25	1.45 (1.01-2.00)
Stomach	69	63	1.09 (0.85-1.38)	14	6	2.37 (1.30-3.98)	21	20	1.06 (0.66-1.63)	34	38	0.90 (0.62-1.26)
Small intestines	11	11	1.01 (0.50-1.81)	1	1	1.20 (0.03-6.67)	2	3	0.66 (0.08-2.40)	8	7	1.14 (0.49-2.24)
Colon	401	371	1.08 (0.98-1.19)	81	31	2.65 (2.11-3.30)	91	107	0.85 (0.69-1.05)	229	234	0.98 (0.86-1.12)
Rectum	171	161	1.06 (0.91-1.24)	16	13	1.19 (0.68-1.93)	56	47	1.20 (0.90-1.55)	99	101	0.98 (0.80-1.20)
Anal canal	18	15	1.17 (0.70-1.85)	5	1	4.40 (1.43-10.26)	6	4	1.45 (0.53-3.16)	7	10	0.70 (0.28-1.43)

Liver	30	31	0.96 (0.65-1.37)	6	3	2.26 (0.83-4.93)	6	9	0.66 (0.24-1.43)	18	19	0.93 (0.55-1.47)
Gallbladder and biliary tract	40	30	1.35 (0.97-1.84)	8	3	3.09 (1.33-6.09)	9	9	1.02 (0.47-1.94)	23	18	1.27 (0.80-1.90)
Pancreas	135	115	1.17 (0.98-1.38)	18	9	1.92 (1.14-3.03)	41	33	1.25 (0.89-1.69)	76	73	1.04 (0.82-1.30)
Smoking-related cancers	827	750	1.10 (1.03-1.18)	137	62	2.19 (1.84-2.59)	216	217	0.99 (0.87-1.14)	474	470	1.01 (0.92-1.10)
Immune-related cancers	168	150	1.12 (0.95-1.30)	34	13	2.59 (1.80-3.62)	44	45	0.98 (0.71-1.32)	90	93	0.97 (0.78-1.20)
Alcohol-related cancers	664	614	1.08 (1.00-1.17)	112	51	2.21 (1.82-2.66)	162	177	0.92 (0.78-1.07)	390	386	1.01 (0.91-1.12)
Obesity-related cancers	867	780	1.11 (1.04-1.19)	145	65	2.23 (1.88-2.62)	225	226	0.99 (0.87-1.13)	497	489	1.02 (0.93-1.11)

O: observed events; E: expected events; SIR: standardized incidence ratios
