# Supplementary material for: Hyperthyroidism or hypothyroidism and gastrointestinal cancer risk: a Danish nationwide cohort study
Source: Endocr Connect. 2018 Aug 31;7(11):1129–35. doi: 10.1530/EC-18-0258 (PMC6215792; doi:10.1530/EC-18-0258)
Supplement: Supporting Table 3 [file ec-7-1129-t003.pdf]

Supplementary Table 3. SIRs for gastrointestinal cancers in 34,703 patients with struma nodosa toxica diagnosed in Denmark in the period 1978-2013, stratified by time of follow-up. Numbers in parentheses indicates 95% CIs.

**Cancer site**	**Overall**			**<1 year**			**1-5 years**			**>5 years**		
	**O**	**E**	**SIR**	**O**	**E**	**SIR**	**O**	**E**	**SIR**	**O**	**E**	**SIR**
Overall	1,257	1,084	1.16 (1.10-1.23)	194	90	2.14 (1.85-2.47)	345	309	1.12 (1.00-1.24)	718	684	1.05 (0.97-1.13)
Esophagus	47	47	1.00 (0.73-1.33)	10	4	2.68 (1.28-4.92)	12	13	0.92 (0.48-1.61)	25	30	0.82 (0.53-1.22)
Stomach	111	88	1.27 (1.04-1.53)	18	9	2.10 (1.24-3.32)	29	28	1.04 (0.70-1.50)	64	51	1.25 (0.96-1.60)
Small intestines	15	13	1.17 (0.65-1.93)	2	1	1.93 (0.23-6.95)	6	4	1.68 (0.62-3.67)	7	8	0.85 (0.34-1.75)
Colon	536	486	1.10 (1.01-1.20)	75	39	1.91 (1.51-2.40)	151	135	1.11 (0.94-1.30)	310	311	1.00 (0.89-1.11)
Rectum	227	204	1.11 (0.97-1.27)	35	17	2.03 (1.41-2.82)	61	58	1.04 (0.80-1.34)	131	128	1.02 (0.85-1.21)
Anal canal	18	17	1.09 (0.64-1.72)	2	1	1.58 (0.19-5.69)	3	4	0.67 (0.14-1.96)	13	11	1.20 (0.64-2.05)

Liver	43	40	1.08 (0.78-1.45)	8	3	2.33 (1.00-4.59)	8	12	0.69 (0.30-1.36)	27	25	1.08 (0.71-1.58)
Gallbladder and biliary tract	46	42	1.11 (0.81-1.48)	7	4	1.84 (0.74-3.80)	16	13	1.27 (0.73-2.07)	23	25	0.91 (0.58-1.37)
Pancreas	214	148	1.44 (1.26-1.65)	37	12	3.04 (2.14-4.19)	59	42	1.42 (1.08-1.83)	118	94	1.25 (1.03-1.50)
Smoking-related cancers	1,135	973	1.17 (1.10-1.24)	175	81	2.16 (1.85-2.51)	312	277	1.13 (1.01-1.26)	648	615	1.05 (0.97-1.14)
Immune-related cancers	233	198	1.17 (1.03-1.33)	37	18	2.04 (1.44-2.82)	62	60	1.03 (0.79-1.33)	134	120	1.11 (0.93-1.32)
Alcohol-related cancers	868	790	1.10 (1.03-1.17)	130	65	2.02 (1.68-2.39)	238	223	1.07 (0.94-1.21)	500	503	0.99 (0.91-1.09)
Obesity-related cancers	1,181	1,014	1.16 (1.10-1.23)	182	85	2.15 (1.85-2.48)	328	289	1.13 (1.01-1.26)	671	640	1.05 (0.97-1.13)

O: observed events; E: expected events; SIR: standardized incidence ratios
