# Supplementary material for: Hyperthyroidism or hypothyroidism and gastrointestinal cancer risk: a Danish nationwide cohort study
Source: Endocr Connect. 2018 Aug 31;7(11):1129–35. doi: 10.1530/EC-18-0258 (PMC6215792; doi:10.1530/EC-18-0258)
Supplement: Supporting Table 4 [file ec-7-1129-t004.pdf]

Supplementary Table 4. SIRs for gastrointestinal cancers in 19,572 patients with other hyperthyroid disease diagnosed in Denmark in the period 1978-2013, stratified by time of follow-up. Numbers in parentheses indicates 95% CIs.

Cancer site	Overall			<1 year			1-5 years			>5 years		
	O	E	SIR	O	E	SIR	O	E	SIR	O	E	SIR
Overall	445	325	1.367 (1.25-1.50)	129	48	2.69 (2.24-3.19)	142	136	1.05 (0.88-1.23)	174	141	1.23 (1.06-1.43)
Esophagus	19	16	1.18 (0.71-1.84)	7	2	2.91 (1.17-5.99)	5	7	0.74 (0.24-1.71)	7	7	1.02 (0.41-2.09)
Stomach	35	23	1.56 (1.08-2.16)	13	4	3.70 (1.97-6.33)	12	10	1.24 (0.64-2.17)	10	9	1.08 (0.51-1.98)
Small intestines	3	4	0.73 (0.15-2.12)	2	1	3.46 (0.42-12.51)	1	2	0.60 (0.02-3.35)	0	2	0.00 (-)
Colon	185	147	1.26 (1.08-1.45)	47	22	2.17 (1.59-2.89)	61	61	0.99 (0.76-1.27)	77	64	1.20 (0.95-1.50)
Rectum	86	62	1.39 (1.11-1.72)	22	9	2.40 (1.50-3.63)	30	26	1.16 (0.78-1.65)	34	27	1.27 (0.88-1.78)
Anal canal	8	5	1.49 (0.64-2.94)	4	1	5.42 (1.47-13.87)	0	2	0.00 (-)	4	2	1.61 (0.44-4.13)
Liver	17	12	1.39 (1.11-1.72)	4	2	2.17 (0.59-5.55)	4	5	0.78 (0.21-1.99)	9	5	1.72 (0.79-3.27)

Gallbladder and biliary tract	16	11	1.49 (0.85-2.42)	1	2	0.62 (0.02-3.47)	5	5	1.11 (0.36-2.59)	10	5	2.16 (1.03-3.97)
Pancreas	76	45	1.70 (1.34-2.12)	29	6	4.47 (2.99-6.42)	24	19	1.29 (0.83-1.92)	23	20	1.16 (0.74-1.73)
Smoking-related cancers	401	292	1.37 (1.24-1.51)	118	43	2.73 (2.26-3.27)	132	122	1.08 (0.90-1.28)	151	127	1.19 (1.01-1.40)
Immune-related cancers	79	55	1.44 (1.14-1.79)	24	8	2.90 (1.86-4.31)	22	23	0.95 (0.60-1.44)	33	24	1.40 (0.96-1.97)
Alcohol-related cancers	310	241	1.28 (1.14-1.43)	82	36	2.30 (1.83-2.85)	101	101	1.00 (0.81-1.22)	127	105	1.21 (1.01-1.44)
Obesity-related cancers	417	303	1.38 (1.25-1.51)	119	45	2.65 (2.20-3.17)	137	127	1.08 (0.91-1.28)	161	131	1.22 (1.04-1.43)

O: observed events; E: expected events; SIR: standardized incidence ratios
